# Strange but True

**Published:** 1877-12-20

**Authors:** 


STRANGE BUT TRUE.—It is a sad commentary upon the intelligence and professional character of medical men in this country, that a very large proportion of them do not take or read a medical journal.

In this age of improvement and discovery, when almost every day something new and important is brought to light, how can a progressive and intelligent man deprive himself of the satisfaction, not to mention the utility, of investigating what is going on in the medical world? What though many things are announced and discussed that do not stand the test of experience, or come up to the full measure of what was originally claimed; yet, is it not true that highly important and valuable additions are being made to the armamentarium of the medical art, and that rapid progress and development is going on in every department of medical science?

It is, perhaps, scarcely credible to some that there are vast numbers of men practicing medicine throughout the country, who know nothing of the proper use and advantages of anæsthetic agents, of chloral, of the bromides, the hypodermic syringe, the speculum, the opthalmoscope, the varied applications of electricity, the varied and important qualities of veratrum, gelseminum, aconite, strychnia, etc., etc. Many of these have not arisen above the old-time routine of calomel, Dover's Powder, and the fly-blister, for any and everything. These are good and useful in their place, but how can a conscientious medical man shut his eyes to other important agents, which have a wider application, and which, indeed, will relieve cases that can not be reached at all by his limited list of remedies. There is a high moral question that can not be overlooked or disregarded by the true physician; and however much he may console himself by the trite and soothing remark, "*I have done all I could*," yet the truth, nevertheless, remains that other means were accessible, of which he was wilfully ignorant, and which might have relieved the patient if they had been used! The mechanic who sets himself up to do good work is justly blamed if he botches the job, whether it be ignorance of the use of the proper tools, or neglect to procure them. So in all other callings and professions. The patient who places his life in your hands, has a legal and moral right to expect that you will give him the benefit of the best and most approved appliances for his relief; and, if you fail to do so, you deceive and wrong him, and do violence to your own conscience.

